# A Simulation Scenario Focused on Resuscitation of a Young Infant (Neonate) for Nurses and Midwives in Malawi, a Limited Resource Country

**DOI:** 10.7759/cureus.2673

**Published:** 2018-05-22

**Authors:** Etta Chimbe Phiri, Bertha Chaputula, Elwin Shawa, Julie Chiaravalli, Elaine Sigalet, Joseph Gabriel, Adam Dubrowski

**Affiliations:** 1 Nursing and Midwifery, Mzuzu University, Mzuzu, MWI; 2 Paediatric, Mzuzu Central Hospital, Mzuzu, MWI; 3 Nursing, Mzuzu Central Hospital, Mzuzu, MWI; 4 Nursing, University of Calgary; 5 Emergency Medicine, Memorial University of Newfoundland, Portugal Cove-St. Philip's, CAN; 6 Emergency Medicine, Pediatrics, Memorial University of Newfoundland, Portugal Cove-St. Philip's, CAN

**Keywords:** resuscitation, young, infant, neonate, nurses, midwives, malawi, simulation, curriculum

## Abstract

Despite the improvement in neonatal and infant mortality rates in Malawi, statistics still identify 27 neonatal mortalities per 1,000 live births and 42 infant mortalities per 1,000 live births. These figures are still unacceptably high. The common causes of neonatal death are prematurity (37%), intrapartum-related complications (28%), and severe infection (24%). These causes create an opportunity to further improve the mortality rates in both populations with a focus on improving the clinical skills of nursing and midwifery students in Malawi. Training to recognize when an infant requires resuscitation, as well as how to perform resuscitative and post-resuscitative care, may help to reduce Malawi’s infant mortality rate in the future.

## Introduction

Mzuzu University is one of the public universities in Malawi that offers a Bachelor of Science in Nursing and Midwifery degree, which includes a formal course in neonatology. In the neonatology course, students are expected to acquire skills in infant resuscitation before proceeding to clinical placements in health facilities. However, the number of students in the program far exceeds the clinical placements at teaching hospitals. This results in limited exposure in a real practice setting. This is further compromised by the limited clinical and educational resources within most of these teaching facilities. Considering these issues, simulation represents a highly useful teaching method to provide nursing and midwifery students at Mzuzu University with opportunities for gaining clinical skills that might otherwise be difficult to obtain.

Simulation is defined as the replication of a task or a clinical event to create an opportunity for students to practice application of their knowledge and skill [1]. Simulation-based medical education (SBME) offers many benefits. It strives to enhance trainees' cognitive, technical, and behavioral skills, as well as self-confidence, with an ultimate benefit for the patient and family being managed. SBME is theoretically supported by the principles of patient safety. It is underpinned by learning theory: the assumption that practicing application of knowledge and skill with feedback will enhance decision making and skill sets which will lead to improved patient outcomes [2]. Per Weller et al. [3], health workers who engage in SBME report greater personal satisfaction, increased knowledge, and improved performance.

To appreciate the goal of engaging SBME in a neonatal and infant care context, it is important to define the population of interest. In Malawi, a young infant is defined as those aged three days up to two months [4-5]. The young infant resuscitation simulation detailed below aims to help learners (who are the in-service health care professionals) to identify when a young infant requires resuscitation, and to give learners the opportunity to practice performing resuscitative and post-resuscitative care in a safe and reproducible setting. In Malawi, most health facilities, including the teaching hospitals, are not equipped with the advanced equipment used to perform resuscitation as it is often done in developed countries. This resuscitation, therefore, aims to prepare learners for real-world practice by employing the same level of basic equipment they will encounter during their time in a Malawi health facility but using locally available simulation technology.

## Technical report

The scenario described in this technical report is designed to support one main and three specific learning objectives. The objectives will be articulated to the learners first, following which the facilitators will use a number of elements to guide the learning: pre-brief, resources and the environment, a scenario stem, the scenario, and the feedback session. After the feedback period, the learners will be given a second opportunity to manage the same scenario again to provide them with the opportunity to implement the feedback. This will help ensure learners will utilize their learning in a real practice setting [3].

Main objective

To create an opportunity for senior university nursing and midwifery learners in limited resource facilities to practice the resuscitation of a young infant with emergency signs.

Specific objectives                   

·      Identify emergency signs using air-breathing circulation (ABC) approach

·      Manage emergency sign before proceeding with rest of systematic assessment.

·      Use team members to help conduct complete assessment and provide timely management.

·      Conduct the resuscitation using bag mask valve (BMV).

·      Conduct the cardiopulmonary resuscitation (CPR).

·      Reassessed ABC.

·      Provide follow-up care after resuscitation (definitive care).

Setting and resources

This simulation session has been designed for Mzuzu University and will be facilitated in the nursing and midwifery skills laboratory.

Resusci® Baby First Aid (model 160-01250) (Laerdal Medical, Stavanger, Norway) is the infant simulator that will be used for these scenarios. The infant simulator cannot breathe or show central cyanosis but can be ventilated with a self-inflating bag. Furthermore, it does not have a pulse and all clinical assessment parameters will be provided to learners by the facilitators in order to increase the realism when using a simulator that is not highly technologically advanced. Therefore, learners will be asked to talk aloud during the assessment and the management of the simulated infant patient so that the facilitators can provide relevant and timely information and prompts. Other equipment that will be needed and is found at the skills laboratory includes an Ambu® bag (Ambu A/S, Ballerup, Denmark), face mask, oxygen concentrator, nasal prongs, nasal catheters, suction catheters, penguin suction devices, and a suction machine.

Process

The entire learning process will occur in two distinct phases. In the first phase, the learners will be familiarized with the technical skills necessary to participate in the simulation scenario. In the second phase, they will be able to practice the scenario using the same simulator, but the focus will be on the development of non-technical skills.

More specifically, in the first phase, the learners have practiced the ABC assessment and management, including oxygen delivery and effective use of a bag and mask to ventilate an infant using a single-handed, two-point top hold technique (referred to as C-E technique because of the “C" shape formation between the thumb and the index finger, and the “E” formation between the remaining three fingers during the hold) [6]. The learners will then be given a brief outline stating the objectives of the simulation scenario prior to the start. The facilitators will demonstrate the technical skills involved in the scenario on the simulator. After the initial demonstrations, the learners will be allowed to practice these skills with guidance until competence is observed by the facilitators.

Next, in the second phase, the learners will be introduced to the entire simulation scenario. The introduction to the scenario will start with a pre-brief, which will take between five and 10 minutes. This is important as it helps create a safe learning environment, familiarizing the learners with expectations and the simulated clinical context [7]. The learners will be oriented to the setting and equipment that will be used in the scenario. They will also be asked to speak out loud and clear so that the facilitator can hear them. They will be encouraged to ask for any information when they feel that the simulated infant cannot provide. Furthermore, the learners will be reminded to maintain a standard of professionalism similar to that of the clinical setting for enhanced learning. Lastly, to create a safe learning environment, the learners will be told that they are expected to make mistakes and it is okay as that is where learning will happen. All learners will be asked to hold the basic assumption that everyone is intelligent, motivated, and cares about doing their best.

Scenario

Table 1 depicts the simulation scenario. The following scenario stem can be used to introduce the learners to the scenario: “Nana, a 7-week-old girl is being admitted to the nursery ward from the "under five outpatient department". The nurse escorting Nana to the ward says he or she is concerned that Nana looks unwell and has started having noisy breathing. Please assess and manage Nana.

**Table 1:** Summary of Simulation Learning Objectives and Expected Actions This table links the learning objectives with the expected actions and potential prompts that the facilitators can use if actions are not executed as expected. ABC: air-breathing circulation; IV: intravenous; SPO_2_: oxygen saturation; CPAP: continuous positive airway pressure

Objective	Expected learner action	Facilitator prompts
Identify emergency signs using ABC approach	Looks for chest rise Listens and feels for airflow Calls for help Gives team member roles (get suction ready, get bag mask ready, get IV access ready)	When the learner says they are looking at the infant, tell them the infant is not crying and has central cyanosis around the mouth If they say they are listening and feeling for airflow, tell them they feel and hear some airflow but there is noise
Manage emergency sign(s) before proceeding with rest of systematic assessment	Positions infant with a roll under shoulders to open the airway Suctions infant (nostril first, then mouth)	Secretions cleared. Airflow unchanged but noise is gone
Use team members to help conduct complete assessment and provide timely management	Assesses breathing, looks for chest rise and respiratory rate Puts saturation monitor on if available Assess heart rate (femoral pulse)	Occasional chest rise and infant has gasping respirations Respiratory rate is 6 breaths per minute Peripheral capillary oxygen saturation (SPO2) is 50% Heart rate is 72 beats per minute (good feedback point as they should not move to C until B is fixed)
Conduct the resuscitation, bag mask valve (BMV)	Puts an appropriately sized mask on the self-inflating bag and connects the bag to the oxygen source Use C-E technique to provide ventilation with bag and mask giving one breath every 3 seconds or after each chest fall [6] Check pulse (femoral)	If the mask is properly sized and technique is good, tell them the saturation starts to rise - 70% with bagging effort - slowly going up After five rescue breaths, tell them the femoral pulse rate is 100 beats/minute If the learner does not initiate BMV or technique is poor, tell them saturation getting worse - 40% to prompt the right action - change to right size mask or change in technique If the learner does not initiate right action and re-assesses young infant after five rescue breaths, tell them; No secretions, airflow no change Hands cool, cap refill 4 seconds and femoral pulse rate 40 beats/minute and weak - to initiate CPR
Conduct the cardiopulmonary resuscitation (CPR)	Alerts team on the need for chest compressions Starts CPR Giving three rescue breaths to one cardiac compression Uses two fingers over sternum just below the nipple line Compresses the chest at least 1/3 diameter of child’s chest If need be, learner bags the child and performs compressions - switch roles after 2 minutes of CPR	If positioning and technique is not correct when learner checks for pulse with compression to determine effectiveness, tell learner there is no improvement, femoral pulse still at 40 beats/minute and weak If positioning and technique is correct, tell learner there is improvement, femoral pulse is 100 bpm - indicating effective compressions
Reassess after every intervention, ABC	Stop compressions to re-assess Checks airway, breathing, and circulation (ABC)	When the learner says they are assessing the ABC, tell them: Airway is clear Spontaneous respirations noted, rise and fall of the chest Respiratory rate increasing - 24 bpm, still some labored breathing and retractions Hands feel cold, capillary refill < 3 seconds and femoral pulse 100
Provide follow-up care after resuscitation (definitive care)	Initiate *b*ubble CPAP (bCPAP) if no contraindications Repeats airway assessment - looks at child activity - listens and feels for airflow Moves to assess breathing: looks for chest rise and work of breathing, listens to breath sounds Moves to assess circulation- hands getting warmer Insert oral gastric tube for feeds Checks temperature and gives paracetamol rectally for fever Gets chest x-ray and full blood count (FBC) and electrolytes Starts antibiotics based on the age of the child Arrange transport to a higher level health facility if at a health centre	bCPAP initiated and saturation increasing slowly Weak cry on and off Airway clear Good airflow Spontaneous chest rise and fall - retractions still present Decreased volume in right lower lobe (RLL) Some retractions Respiratory rate (RR) 40 minute Oxygen saturation on bCPAP 95% Hands warmer, capillary refill < 3 seconds Heart rate 120 beats per minute If the physician/clinician was not around during the resuscitation, then he/she reviews the neonate, may order chest x-ray, laboratory tests, and antibiotics

Feedback

Feedback on learners’ performance is the most important feature of simulation education as it creates an opportunity for guided personal reflection about their performance and insight into performance gaps that may not be obvious to them [8]. The goal of providing feedback is to close the gaps to improve management and simulated patient outcomes. In SBME, learners may get emotional because of something they did or wish they did in front of their peers, or a perception that they would have done things differently in a real situation as the information and clinical findings in real patients would have provided more accurate guidance. Giving learners an opportunity to express their emotions is helpful as it allows them to focus on discussion and learning. If learners respond defensively, the facilitator should address their concerns in a non-confrontational way. The learners’ responses and details about what frustrated them often provide the facilitator with important insight into the needs of the learners, inclusive of how the SBME might be improved to support more realism. Once emotion is expressed, the facilitator can focus on feedback.

Three methods of giving feedback can be used for the scenario, and the choice of the method will depend on the learners’ and facilitators’ experiences with simulation [7]. These include:

1. Direct feedback: A facilitator-centered feedback method that focuses on closing any performance gaps by telling the learner what they have done wrong and how to do it appropriately next time. 

2. Plus/delta: A learner-centered feedback method that focuses on the learners identifying what they did well, what potential performance gaps challenged management (what would they do differently next time), and elaborate on how to close these gaps. In this method, the facilitators may use frames such as "What worked well? What would you do again?” to identify the positive aspects of performance (i.e. the plus). This is typically followed by phrases such as "What would you do differently?” to identify the perceived gaps in performance that need to be changed (i.e., the delta). Finally, the facilitators can check if the performance gaps have been closed by introducing a phrase to stimulate reflection such as "How would you summarize your experience? What did you learn?”.

3. Observation, point of view, and inquiry: A learner-centered feedback method that is used to check the learners' assumptions and to ensure that the feedback is based not only on learners' perceptions of what happened but also why actions were taken (i.e., the assumptions). In this method, the facilitator identifies what they saw or did not see and heard or did not hear. After that, they express their concern about these points and then direct it back to the learners to get their perspectives. This approach focuses on identifying thoughts or experiences that led to learner actions so they can be discussed further and performance gaps can be closed.

## Discussion

The technical report described here is a direct outcome of a larger initiative looking at the development of simulation expertise in developing countries described in our earlier paper [7]. The long-term aim of our initiative is to decrease morbidity and mortality through the introduction of a context-specific faculty development program focused on pedagogy and the administration of SBL programs. In this regard, we have successfully developed and implemented several train-the-trainer courses described earlier [7]. We hypothesized that the knowledge developed through these courses will cascade in a few directions – from new courses being offered across Malawi in health institutions to individual educators taking initiative and writing technical reports describing locally relevant simulation scenarios [9].

## Conclusions

The simulation scenario described in this technical report was designed to improve the ability of senior nursing and midwifery university students in Malawi to recognize when an infant requires resuscitation, perform effective resuscitation, and provide post-resuscitative care to help reduce Malawi’s infant mortality rate in the future.

It is our intention that by sharing this scenario, it may be used in other such settings with nursing and midwifery students to build their capacity with neonatal emergency management. From our experience, the inclusion of SBME creates opportunities for learning in circumstances when learning cannot be achieved in the real practice setting. It also decreases potential risk to real patients as midwives and nurses responding to these challenging events are confident and competent because they have practiced the management. Ultimately, SBME creates an opportunity to improve how we care for this vulnerable population, which should positively impact these high mortality rates over time. 

In the future, the simulation scenario described in this technical report will be embedded into the training schedule at the university and affiliated teaching hospitals in order to assess its effectiveness in achieving the learning objectives.
